# Supplementary material for: Metformin abrogates pathological TNF-α-producing B cells through mTOR-dependent metabolic reprogramming in polycystic ovary syndrome
Source: eLife. 2022 Jun 24;11:e74713. doi: 10.7554/eLife.74713 (PMC9270024; doi:10.7554/eLife.74713)
Supplement: Figure 1—source data 1. [file elife-74713-fig1-data1.pdf]

Figure 1. TNF-α production by pathological B cells in women with PCOS.

A, Serum TNF-α concentration

Control	PCOS	PCOS
23.59	45.23	47.87
28.76	53.24	39.66
34.3	45.93	29.92
25.61	26.8	35.61
38.01	42.19	54.44
15.98	15.76	52.73
33.36	42.19	27.59
37.83	57.33	17.08
35.05	46.29	47.87
41.65	23.18	48.04
20.29	59.51	38.93
49.61	41.83	43.45
21.54	26.00	/

B, TNF-α expression in $CD19^+$ B cells was determined by western blot

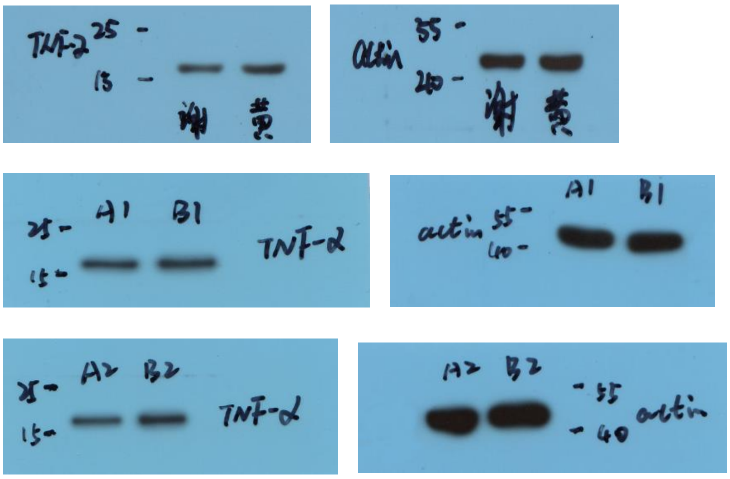

Control- TNF-α/β actin	PCOS- TNF-α/β actin
0.24	0.36
0.15	0.26
0.24	0.4

C, Percentage of TNF-α^+ cells in $CD19^+$ B cells

Control	PCOS	PCOS
4.9	13.6	14.6
7.3	9.5	16
10.5	8.8	13
6.1	15.8	12
5.6	11.9	8.7
5.2	18.8	14.3

7.5	11.7	17.2
/	/	7.9

D, TNF-α in the culture supernatants (sup.)

Control	PCOS
105.16	206.77
171.16	297.72
163.26	292.73
152.41	/

E, the correlation between the percentage of TNF-α$^+$ cells in CD19$^+$ B cells and serum AMH levels by Pearson's correlation analyses.

TNF-α$^+$ cells in CD19$^+$ B cells (%)	4.9	7.3	10.5	6.1	5.6	5.2	7.5	13.6	9.5	/
AMH	1.99	10.51	3.34	4.16	10.8	1.74	1.78	6.56	22.73	/
TNF-α$^+$ cells in CD19$^+$ B cells (%)	8.8	15.8	11.9	18.8	11.7	16	13	8.7	17.2	7.9
AMH	8.03	15.61	7.77	15.49	10.46	9.94	9.71	5.67	12.82	7.41

F, IL-6, IL-8, IFN-γ, TNF-α, CCL2 and CCL20 mRNAs expression levels in granulosa cells were measured by qPCR.

**IL-6**		**IL-8**		**IFN-γ**		**TNF-α**		**CCL2**		**CCL20**	
Control	TNF-α	Control	TNF-α	Control	TNF-α	Control	TNF-α	Control	TNF-α	Control	TNF-α
1	12.87	1	43.28	1	0.45	1	1.31	1	23.35	1	13.13
2.23	9.35	1.53	35.26	1.05	3.46	0.61	3.57	0.55	6.06	0.69	6.66
1	3.46	1	23.02	1	1.38	1	1.43	1	3.69	1	2.81
3.81	5.11	1.46	26.72	1.43	1	1.08	0.42	1.58	1.34	1.56	1.59
1	6.66	1	35.63	1	0.46	1	1.24	1	6.59	1	6.54
1	5.56	1	16.51	1	0.18	1	1.9	1	3.96	1	4.36
1.75	28.44	0.71	36.38	2.23	1.32	1.78	1.28	1.11	5.66	1.08	5.74
