# Supplementary material for: Metformin abrogates pathological TNF-α-producing B cells through mTOR-dependent metabolic reprogramming in polycystic ovary syndrome
Source: eLife. 2022 Jun 24;11:e74713. doi: 10.7554/eLife.74713 (PMC9270024; doi:10.7554/eLife.74713)
Supplement: Figure 2—source data 1. [file elife-74713-fig2-data1.pdf]

Figure 2. Metformin inhibits TNF-α production by pathological B cells in PCOS.

A, TNF-α expression in stimulated B cells from women with PCOS with or without metformin.

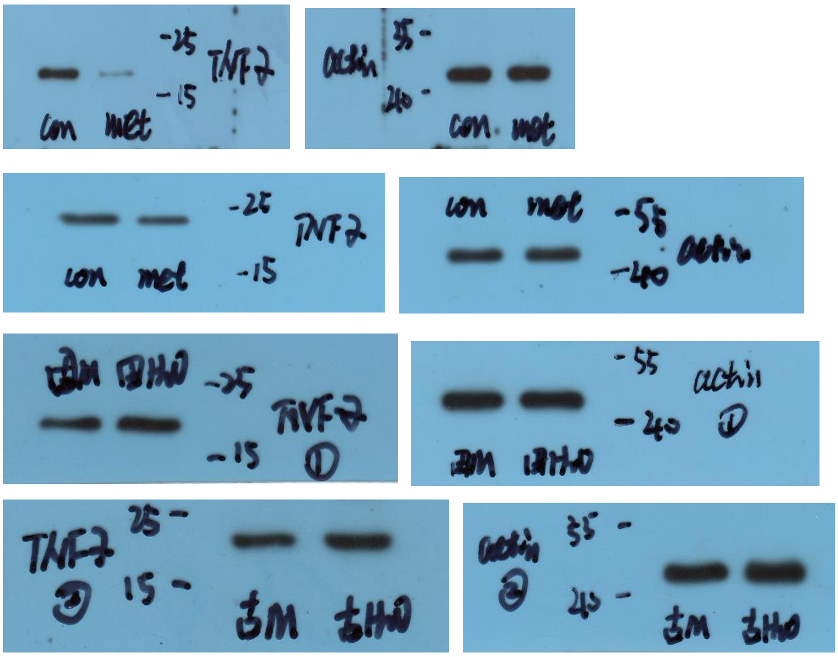

B cells TNF-α/β actin	B cells+Met- TNF-α/β actin
0.55	0.32
0.4	0.28
0.61	0.06
0.32	0.18

B, Percentage of TNF-α^+ cells in $CD19^+$ B cells

B cells	B cells+Met
10.4	2.7
8	0.6
8.3	2.3
11.5	3.4
10	0.7
5.8	0.2

C, TNF-α mRNA expression levels in B cells

B cells	B cells+Met
1	1.12
1.83	2.67
1.37	1.61

1	1.19
1.35	0.28
0.61	1.22

D, Percentage of TNF-α^+ cells in CD19$^+$ B cells from women with PCOS, before (pre) and after (post) treatment with metformin

Pre Met	Post Met
10.8	7.4
12	8.6
9.1	7.4

Fig supplement 1: **Metformin does not affect B cell proliferation and apoptosis.**

B-D, The ratios of alive, early apoptotic and dead cells in B cells by flow cytometry

alive cells		early apoptotic cells		dead cells	
B cells	B cells+Met	B cells	B cells+Met	B cells	B cells+Met
75.2	81.8	6.4	5.3	11.4	8.2
71	66.3	10	11	13.1	15.3
85.1	86.8	6.2	5.9	7.4	6.4
77.6	79	10.9	9.9	10.1	10
