# Supplementary material for: Metformin abrogates pathological TNF-α-producing B cells through mTOR-dependent metabolic reprogramming in polycystic ovary syndrome
Source: eLife. 2022 Jun 24;11:e74713. doi: 10.7554/eLife.74713 (PMC9270024; doi:10.7554/eLife.74713)
Supplement: Figure 3—source data 1. [file elife-74713-fig3-data1.pdf]

Figure 3. Metformin inhibits mTOR phosphorylation in pathological B cells.

A, phosphorylated (P-) AMPK (S487)/AMPK, P-PI3K p85 (Tyr458)/PI3K p85 and P-mTOR (S2481)/ mTOR in B cells between women with PCOS and control subjects.

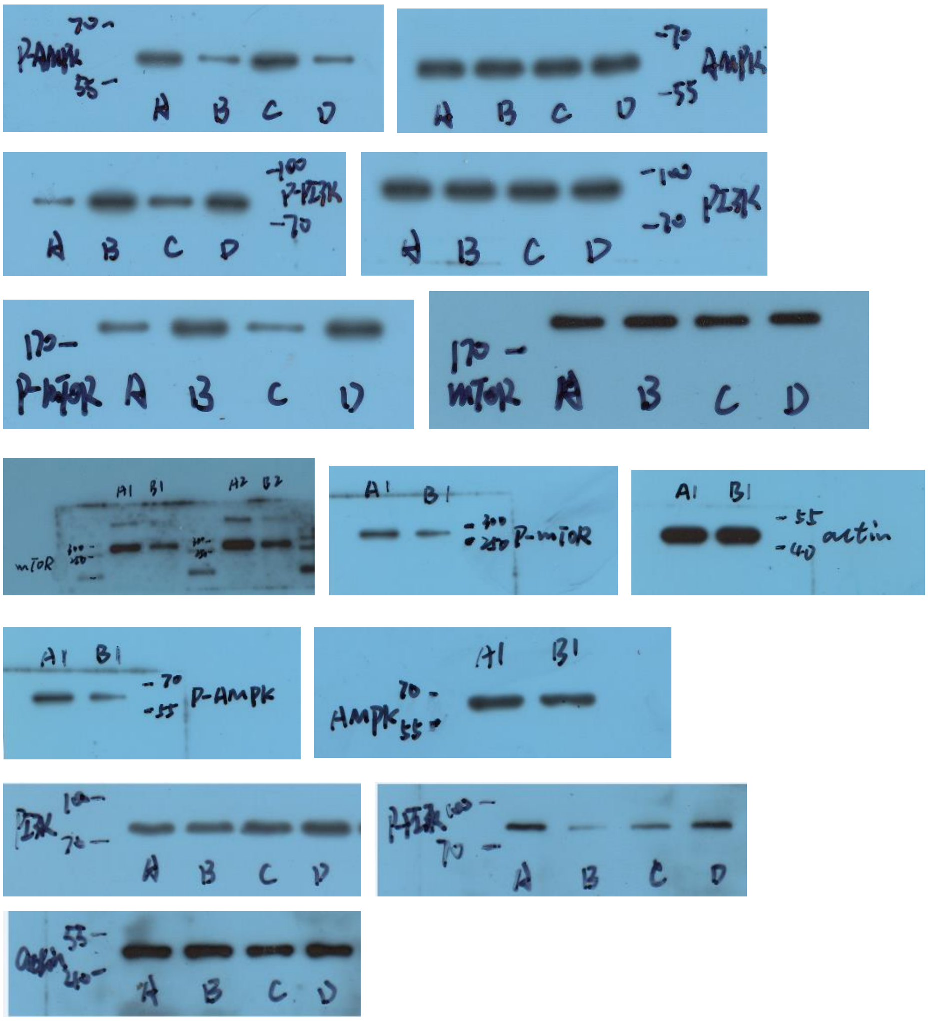

B-D, quantification analysis of the ratios of phosphorylated (P-) AMPK (S487)/AMPK, P-PI3K p85 (Tyr458)/PI3K p85 and P-mTOR (S2481)/ mTOR in B cells.

P-AMPK (S487)/AMPK		P-PI3K p85 (Tyr458)/PI3K p85		P-mTOR (S2481)/ mTOR	
Control	PCOS	Control	PCOS	Control	PCOS
0.4	0.08	0.26	0.51	0.17	0.69
0.81	0.11	0.04	0.68	0.1	0.81
0.43	0.2	0.08	0.57	0.25	0.4

E, P-AMPK (S487)/AMPK, P-PI3K p85 (Tyr458)/PI3K p85 and P-mTOR (S2481)/ mTOR in stimulated B cells from women with PCOS with or without metformin.

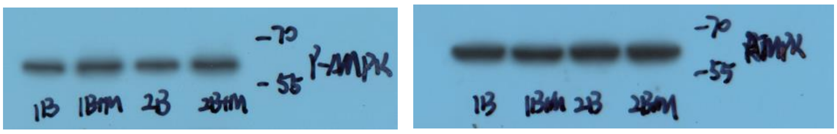

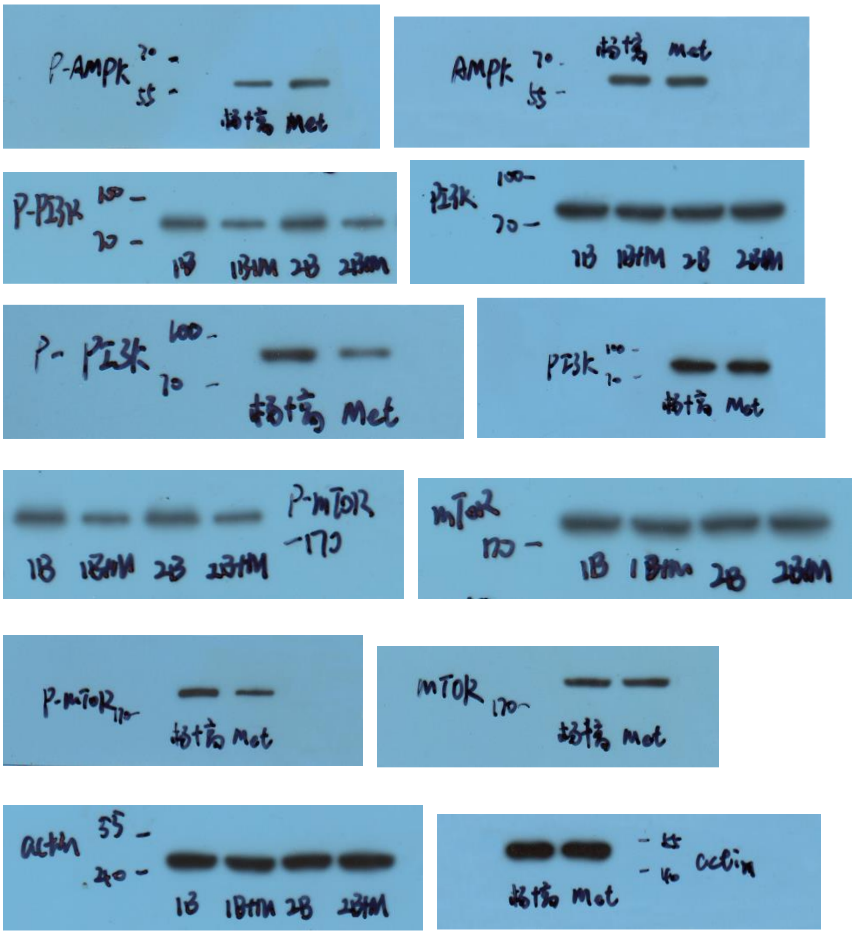

F-H, quantification analysis of the ratios of P-AMPK (S487)/AMPK, P-PI3K p85 (Tyr458)/PI3K p85 and P-mTOR (S2481)/ mTOR in stimulated B cells from women with PCOS with or without metformin.

P-AMPK (S487)/AMPK		P-PI3K p85 (Tyr458)/PI3K p85		P-mTOR (S2481)/ mTOR	
B cells	B cells+Met	B cells	B cells+Met	B cells	B cells+Met
0.33	0.46	0.41	0.17	0.81	0.38
0.31	0.5	0.65	0.21	0.7	0.36
0.27	0.48	0.53	0.18	0.75	0.45

I, P-mTOR (S2481)/ mTOR in B cells isolated from women with PCOS, before (pre) and after (post) treatment with metformin.

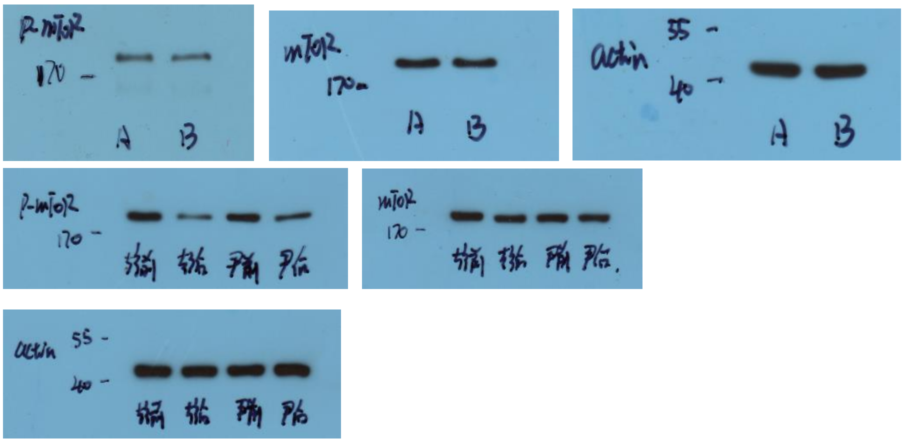

J, quantification analysis of the ratio of P-mTOR (S2481)/ mTOR in B cells isolated from PB of women with PCOS, before (pre) and after (post) treatment with metformin

Pre Met	Post Met
0.75	0.22
0.72	0.48
0.7	0.45
