# Supplementary material for: Metformin abrogates pathological TNF-α-producing B cells through mTOR-dependent metabolic reprogramming in polycystic ovary syndrome
Source: eLife. 2022 Jun 24;11:e74713. doi: 10.7554/eLife.74713 (PMC9270024; doi:10.7554/eLife.74713)
Supplement: Figure 4—source data 1. [file elife-74713-fig4-data1.pdf]

Figure 4. Metformin induces mitochondrial remodeling in pathological B cells.

A, Percentage of TNF-α$^+$ cells in CD19$^+$ B cells from women with PCOS stimulated with B cell-targeted activator with or without oligomycin A.

B cells	B cells+ Olig A
9.6	5.3
19.8	6.7
11.6	8.4
14.4	8.3
14.3	6.9

C, The length/width ratio of per mitochondria in B cells

B cells	1.69	1.75	2.8	1.15	5	3.76	1.29	1.51
B cells+Met	1.06	1.29	1.04	1.38	1.12	1.29	1.27	1.22
B cells	1.54	3.02	2.08	1.01	3.29	1.6	1.44	2.25
B cells+Met	1.51	1.17	1.4	1.15	1.21	1.04	1.22	1.3

D, The numbers of mitochondria in per B cell

B cells	5	8	5	3	9	6	5	9	10	9	7
B cells+Met	7	5	3	6	5	5	7	6	4	7	6

E-G, MMP, mitochondrial mass and ROS was measured

MMP		Mitochondrial mass		ROS	
B cells	B cells+Met	B cells	B cells+Met	B cells	B cells+Met
1	0.92	1	0.98	1	0.17
1	0.94	1	1.07	1	0.23
1	0.82	1	1.01	1	0.19
1	0.75	1	1.07	1	0.1
1	0.9	1	0.94	/	/
