# Supplementary material for: Metformin abrogates pathological TNF-α-producing B cells through mTOR-dependent metabolic reprogramming in polycystic ovary syndrome
Source: eLife. 2022 Jun 24;11:e74713. doi: 10.7554/eLife.74713 (PMC9270024; doi:10.7554/eLife.74713)
Supplement: Figure 5—source data 1. [file elife-74713-fig5-data1.pdf]

**Figure 5. Metformin reduces glucose uptake in pathological B cells.**

A, Glucose uptake was measured

| | | | | | | | |
|---|---|---|---|---|---|---|---|
| B cells | 1 | 1 | 1 | 1 | 1 | 1 | 1 |
| B cells+Met | 0.63 | 0.53 | 0.62 | 0.92 | 0.96 | 0.95 | 0.57 |

B-D, Glut 1 and Glut 4 expression in CD19$^{+}$ B cells by western blot

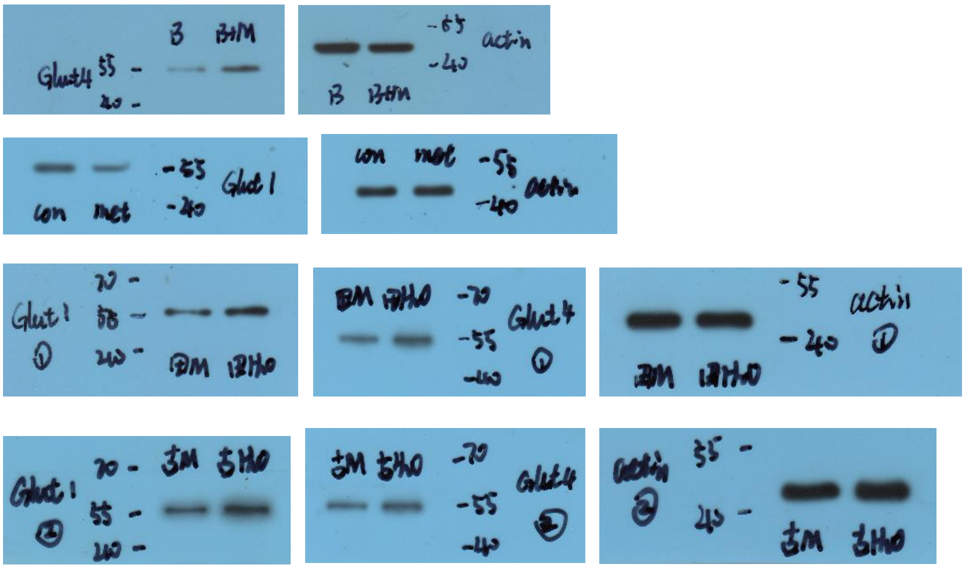


| Glut 1/β actin | | Glut 4/β actin | |
|---|---|---|---|
| B cells | B cells+Met | B cells | B cells+Met |
| 0.32 | 0.1 | 0.33 | 0.1 |
| 0.37 | 0.13 | 0.23 | 0.13 |
| 0.37 | 0.14 | 0.32 | 0.05 |

E-F, Percentage of Glut 1$^{+}$ cells and Glut 4$^{+}$ cells in CD19$^{+}$ B cells

| | Glut 1$^{+}$ cells-B cells | | | | | | | | Glut 4$^{+}$ cells-B cells | | | | | | | |
|---|---|---|---|---|---|---|---|---|---|---|---|---|---|---|---|---|
| 30min | 2.4 | 1.1 | 1.2 | 0.7 | 1.5 | 1.9 | / | / | 8.6 | 11.4 | 11.1 | 12.3 | 5.9 | 8.1 | 4.8 | 8.6 |
| 4h | 9.5 | 4.7 | 4.3 | 4.1 | 3 | 2 | 1.8 | 5.7 | 0.3 | 0.5 | 0.7 | 1.1 | 0.2 | 0 | / | / |
| | Glut 1$^{+}$ cells -B cells+Met | | | | | | | | Glut 4$^{+}$ cells- B cells+Met | | | | | | | |
| 30min | 2.4 | 1.2 | 1.1 | 0.3 | 1.5 | 0.5 | / | / | 3 | 8.5 | 10.6 | 3.5 | 4.2 | 6 | 5 | / |
| 4h | 7.9 | 3.4 | 2 | 2.9 | 3.4 | 2.4 | 1.4 | 4 | 1.2 | 0.5 | 0.8 | 1.2 | 0.5 | 0.2 | / | |

G-I, HIF1α and c-Myc expression in CD19$^{+}$ B cells by western blot

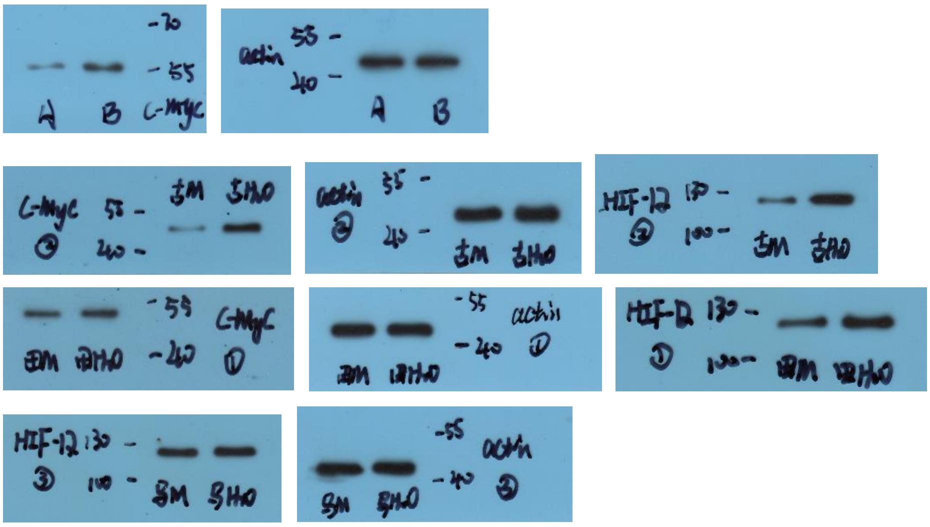

| HIF1α /β actin | | c-Myc /β actin | |
|---|---|---|---|
| B cells | B cells+Met | B cells | B cells+Met |
| 0.55 | 0.22 | 0.32 | 0.2 |
| 0.53 | 0.09 | 0.35 | 0.03 |
| 0.51 | 0.37 | 0.39 | 0.09 |
