# Supplementary material for: Metformin abrogates pathological TNF-α-producing B cells through mTOR-dependent metabolic reprogramming in polycystic ovary syndrome
Source: eLife. 2022 Jun 24;11:e74713. doi: 10.7554/eLife.74713 (PMC9270024; doi:10.7554/eLife.74713)
Supplement: Figure 6—source data 1. [file elife-74713-fig6-data1.pdf]

Figure 6. Rapamycin inhibits TNF-α production, induces mitochondrial remodeling and reduces glucose uptake in pathological B cells.

A, Percentage of TNF-α^+ cells in CD19$^+$ B cells

B cells	B cells+ Rapa 5um-24h	B cells+ Rapa 5um-48h
14.3	5.3	1.4
12.5	6.1	1.2
17.2	4.1	1.7
7.9	/	/
12.5	/	/
15.3	/	/

B-E, MMP, mitochondrial mass, ROS and 2-NBDG was measured

MMP		Mitochondrial mass		ROS		2-NBDG	
B cells	B cells+Rapa	B cells	B cells+Rapa	B cells	B cells+Rapa	B cells	B cells+Rapa
1	0.48	1	0.99	1	0.36	1	0.74
1	0.58	1	0.87	1	0.31	1	0.68
1	0.51	1	1.12	1	0.39	1	0.52
