# Supplementary material for: Metformin abrogates pathological TNF-α-producing B cells through mTOR-dependent metabolic reprogramming in polycystic ovary syndrome
Source: eLife. 2022 Jun 24;11:e74713. doi: 10.7554/eLife.74713 (PMC9270024; doi:10.7554/eLife.74713)
Supplement: Figure 7—source data 1. [file elife-74713-fig7-data1.pdf]

Figure 7. Metformin treatment improves the PCOS pathological phenotypes in DHEA-induced mice.

C-D, Quantitative analysis of cystic follicles and corpora lutea.

cystic follicles			corpora lutea		
Control	DHEA	DHEA+Met	Control	DHEA	DHEA+Met
0	3	1	7	1	5
0	2	1	6	3	2
0	3	1	8	3	6
0	2	3	7	0	4
0	0	0	7	1	3
0	4	4	4	2	6

F, Glucose tolerance test (GTT)

	Control						DHEA						
0 min	4.4	4	4.3	4.5	4.3	4.1	3.9	3.3	4.2	3.5	3.5	3.5	3.5
15 min	16.5	17.3	16.8	14.7	16.7	16.5	24.2	28.8	17.2	25.8	24.7	20.9	22.2
30 min	9.5	12	9.8	8.6	11.6	8.4	22.8	23.3	14.9	26.7	11.7	21.5	27
60 min	8.3	7.7	7.9	6.8	8.6	8.3	11.8	15	6.7	13.1	8.6	9.7	16
90 min	7.1	7.3	7.4	6.7	7.3	6.5	8.8	6.9	5.2	11.7	5.6	7.8	7.3
120 min	6.7	6.1	7.3	5.7	5.8	6.2	6.4	8.7	3.3	5.5	4.1	7	6.5
	DHEA+Met												
0 min	2.8	3	4	2.9	3.4	2.8							
15 min	13.2	20.5	15.5	12.4	13.6	17.9							
30 min	7.4	15.7	13	17.4	13.3	13.3							
60 min	5.9	8.6	8.6	10.5	7.3	6.8							
90 min	5.8	5.9	5.6	6.1	5	7.1							
120 min	5.1	5.2	5.8	5.9	5.4	5.6							

G, GTT area under the curve

Control	DHEA	DHEA+Met
1057	1619	813
1101	1769	1196
1073	1031	1068

938.3	1841	1186
1108	1148	978.8
1004	1454	1090
/	1763	/

H, Serum TNF-α concentrations

Control	DHEA	DHEA+Met
4.33	14.72	5.99
3.4	13.82	7.15
4.33	9.65	8.6
3.41	10.32	6.77
3.88	11.63	6.38
/	6.77	/
