# Supplementary material for: Metformin abrogates pathological TNF-α-producing B cells through mTOR-dependent metabolic reprogramming in polycystic ovary syndrome
Source: eLife. 2022 Jun 24;11:e74713. doi: 10.7554/eLife.74713 (PMC9270024; doi:10.7554/eLife.74713)
Supplement: Figure 8—source data 1. [file elife-74713-fig8-data1.pdf]

Figure 8. Metformin inhibits TNF-α production, alters AMPK/PI3K/mTOR phosphorylation and induces metabolic reprogramming in pathological B cells from DHEA-induced PCOS mice.

A, Percentage of TNF-α^{+} cells in splenic CD19^{+} B cells

Control	2.6	1.5	1.5	3.1	2.5	/	/	/	/
DHEA	11.4	5.7	7.4	5.7	11.3	5.5	5.7	/	/
DHEA+Met	0.5	1.6	2.6	7.7	6	4.9	2	0.6	1.4

B, P- AMPK (S487)/AMPK, P-PI3K p85 (Tyr458)/PI3K p85 and P-mTOR (S2481)/ mTOR in splenic B cells between DHEA-induced PCOS mice and controls.

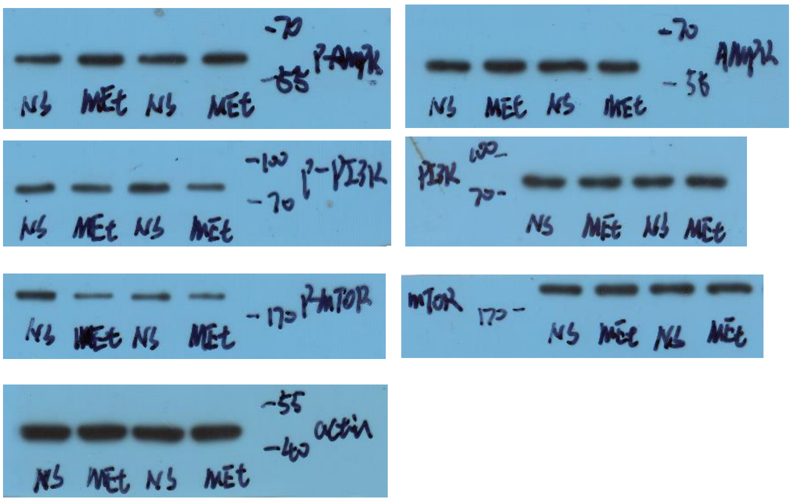

D, Differential energy metabolites were quantitatively analyzed

Lactate			Succinate			Fumarate		
Control	DHEA	DHEA+Met	Control	DHEA	DHEA+Met	Control	DHEA	DHEA+Met
173.0904	158.8494	383.5425	8.4587	6.6328	14.285	1.3645	0.3066	2.0179
250.2598	240.5514	438.3095	9.0338	8.0549	16.447	0.8652	1.349	2.9449
255.6909	262.1899	520.6824	5.0388	10.2961	20.7508	0.7142	0.7558	3.1855

L-Malic acid			Oxaloacetate		
Control	DHEA	DHEA+Met	Control	DHEA	DHEA+Met
5.8868	9.5513	8.9067	74.9818	57.0697	108.1906
5.7234	7.991	11.2807	84.9568	57.6216	112.5176
5.2725	6.3785	14.3058	58.2695	87.096	139.9998
